# COI metabarcoding of large benthic Foraminifera: Method validation for application in ecological studies

**DOI:** 10.1002/ece3.9549

**Published:** 2022-11-22

**Authors:** Elsa B. Girard, Jan‐Niklas Macher, Jamaluddin Jompa, Willem Renema

**Affiliations:** ^1^ Naturalis Biodiversity Center Leiden the Netherlands; ^2^ IBED University of Amsterdam Amsterdam the Netherlands; ^3^ Marine Science Department, Faculty of Marine Science and Fisheries Hasanuddin University Makassar Indonesia

**Keywords:** bulk‐sample DNA, community composition, coral reef, environmental DNA, foraminifera, metabarcoding

## Abstract

Monitoring community composition of Foraminifera (single‐celled marine protists) provides valuable insights into environmental conditions in marine ecosystems. Despite the efficiency of environmental DNA (eDNA) and bulk‐sample DNA (bulk‐DNA) metabarcoding to assess the presence of multiple taxa, this has not been straightforward for Foraminifera partially due to the high genetic variability in widely used ribosomal markers. Here, we test the correctness in retrieving foraminiferal communities by metabarcoding of mock communities, bulk‐DNA from coral reef sediment samples, and eDNA from their associated ethanol preservative using the recently sequenced cytochrome c oxidase subunit 1 (COI) marker. To assess the detection success, we compared our results with large benthic foraminiferal communities previously reported from the same sampling sites. Results from our mock communities demonstrate that all species were detected in two mock communities and all but one in the remaining four. Technical replicates were highly similar in number of reads for each assigned ASV in both the mock communities and bulk‐DNA samples. Bulk‐DNA showed a significantly higher species richness than their associated eDNA samples, and also detected additional species to what was already reported at the specific sites. Our study confirms that metabarcoding using the foraminiferal COI marker adequately retrieves the diversity and community composition of both the mock communities and the bulk‐DNA samples. With its decreased variability compared with the commonly used nuclear 18 S rRNA, the COI marker renders bulk‐DNA metabarcoding a powerful tool to assess foraminiferal community composition under the condition that the reference database is adequate to the target taxa.

## Graphical abstract

We test the correctness in retrieving foraminiferal communities by metabarcoding of mock communities, bulk‐DNA from coral reef sediment samples, and eDNA from their associated ethanol preservative using the recently sequenced cytochrome c oxidase subunit 1 (COI) marker. We compared our results to large benthic foraminiferal communities previously reported from the same sampling sites and found that bulk‐DNA metabarcoding of large benthic foraminifera showed a significantly high species richness and also detected additional species. Our study confirms that metabarcoding using the foraminiferal COI marker adequately retrieves the diversity and community composition of both the mock communities and bulk‐DNA samples.

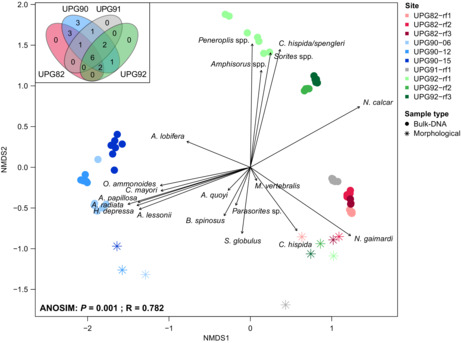

## 1 INTRODUCTION

Monitoring the community composition of Foraminifera provides valuable insights into environmental conditions in marine ecosystems (Frontalini et al., 2018; Frontalini & Coccioni, 2008; Oladi & Shokri, 2021; Oliver et al., 2014). Foraminifera, marine protists, are major actors in the carbonate budget of the ocean, accounting for up to 70% of the sediments (Renema, 2006; Tudhope & Scoffin, 1988). Large benthic symbiont‐bearing foraminifera (LBF), especially, have been identified as good bioindicators of coral reef health (Cockey et al., 1996; Hallock, 1996; Prazeres et al., 2020), with the potential to foreshadow decreasing conditions to coral growth and further reef degradation (Girard, Estradivari, et al., 2022).

Foraminiferal community compositions are currently most reliably assessed through time‐consuming manual sorting and classification by microscopy. In recent years, metabarcoding methods using environmental DNA (eDNA) and bulk‐sample DNA (bulk‐DNA) have been shown to be powerful tools for monitoring the presence of multiple taxa (van der Loos & Nijland, 2020 and references herein), including Foraminifera (e.g., Al‐Enezi et al., 2022; Barrenechea Angeles et al., 2020; Frontalini et al., 2018, 2020; He et al., 2019; Laroche et al., 2016; Morard et al., 2016; Pawlowski et al., 2016; Weber & Pawlowski, 2013). Assessing foraminifera communities with bulk‐ and eDNA metabarcoding has shown multiple advantages. First, assuming the availability of an adequate reference database, it can reduce misclassification of species that have high morphological similarities and increase resolution in detected taxa (Frontalini et al., 2018). Additionally, metabarcoding workflows are efficient and time‐effective, contrary to manually assessing foraminiferal community compositions (Frontalini et al., 2020; Weber & Pawlowski, 2013). However, using metabarcoding methods has not been straightforward, first, due to the high genetic variability in the widely used nuclear 18 S ribosomal RNA marker and the difference in gene copy number between species (Girard, Langerak, et al., 2022; Milivojević et al., 2021; Morard et al., 2016, 2018; Pillet et al., 2012; Weber & Pawlowski, 2013, 2014). Second, gaps in the available reference database are another limitation, although a lot of effort has been spent on the ribosomal reference library (ca. 1100 nonredundant species sequences; Frontalini et al., 2018; He et al., 2019); about 9600 species of extant foraminifera are reported in the World Foraminifera database (Hayward et al., 2020). Third, taxonomic composition between morphology‐based and DNA‐based datasets can be different, mainly because test‐bearing foraminifera are easier to identify using morphological characters than species without a test (Frontalini et al., 2020). However, diversity metrics were observed to be congruent between morphology‐based and DNA‐based datasets (Frontalini et al., 2020).

The issues related to the use of ribosomal markers might be reduced using a different marker in combination. Recently, Macher, Wideman, et al. (2021) successfully amplified the cytochrome c oxidase subunit 1 (COI) in Foraminifera. The COI marker happens to be conserved within Foraminifera and its genetic variability is low in, at least, some species of the orders Rotaliida and Miliolida (Girard, Langerak, et al., 2022). Therefore, COI metabarcoding might help to adequately assess foraminiferal community compositions and generate data for environmental monitoring of different marine ecosystems. Besides this, an enlarged COI reference database is needed for a reliable annotation of resulting metabarcoding sequences. Macher et al. (2022) recently published a proof of concept of COI metabarcoding for Foraminifera, performed on beach sand samples from the North Sea. Here, we validate the method by comparing results from foraminiferal COI metabarcoding of six mock communities, bulk‐DNA from 10 reef sediment samples, and eDNA from their associated ethanol preservative. The annotation of sequences to foraminiferal taxa was based on our self‐built COI reference database from 77 morphospecies. We assess the detection success of the metabarcoding workflow on the bulk‐DNA and eDNA samples with results of LBF communities reported by Girard, Estradivari, et al. (2022).

## 2 METHODS

### 2.1 Sediment sampling and mock community preparation

**FIGURE 1:**
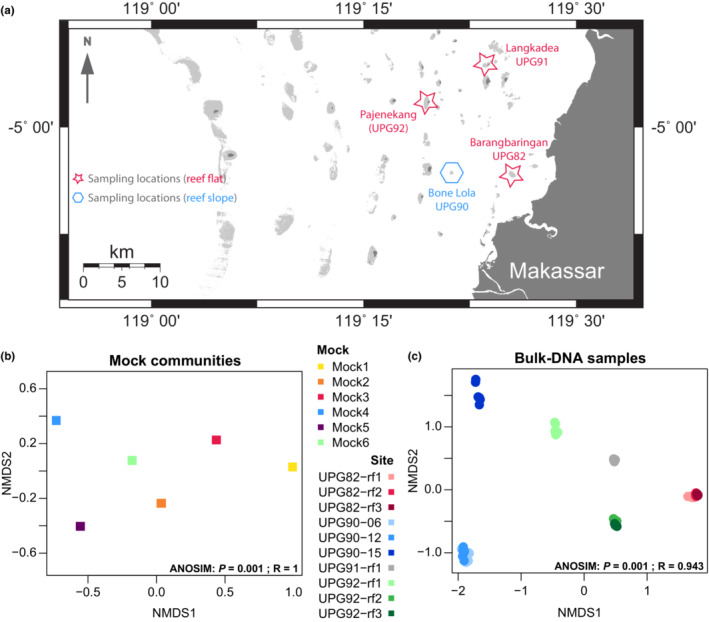
(a) Map of the sampling locations in the Spermonde Archipelago, South‐West Sulawesi, Indonesia. (b, c) non‐metric dimensional scaling representation of the mock communities (b) and the bulk‐DNA samples from the sediment alone (c), including all their replicates and all ASVs. Analysis of similarity (ANOSIM) results are indicated at the lower right corner of each graph (b: grouping per mock community; c: grouping per site). Mock communities and sampling sites are marked by different colors. Each mock community has five technical replicates, which overlap each other in the graph (b).

In April–May 2018, 10 sediment samples were collected on the reef flat around the islands Barangbaringan (UPG82), Langkadea (UPG91), Pajenekang (UPG92) and on the reef slope around the island Bone Lola (UPG90) in the Spermonde Archipelago (Southwest Sulawesi, Indonesia) (see Table S1, Figure 1a). At each sampling location, between one and three sites were sampled. The samples were taken by sampling a circular surface of approximately 1000 cm^2^ of the substratum into sampling bags by hand or using a small trowel. A subsample of each sample was preserved in a 8‐ml twist‐cap tube with 96% ethanol rapidly after sampling for molecular analysis and transported back to the Naturalis Biodiversity Center (NBC), the Netherlands. Ethanol was used as fixative to ease the sample transportation from Indonesia to NBC without needing to keep the samples frozen at all time. These samples were then stored at −20°C at NBC until DNA extraction in November 2021.

Additionally, six mock communities were prepared by combining eight or nine different species per mock community, where each species was represented by one specimen in a clean 1.5‐ml eppendorf tube (see Table S2 for mock community composition). The selected species were LBF from the order Rotaliida and Miliolida collected in the Spermonde Archipelago during the same field campaign, as stated above. Before adding the specimens in the different mock communities, we cleaned their shells with a brush and photographed each specimen using a stereomicroscope mounted with a camera (Leica, Wetzlar, Germany).

### 2.2 DNA extraction

To extract bulk‐sample DNA (bulk‐DNA) from the sediment samples, we removed (and reserved at 4°C) the ethanol and dried the samples in an oven at 55°C overnight. The sediment samples were then crushed to coarse powder with a mortar and pestle. The mortar and pestle were thoroughly rinsed in sequence with ethanol 70%, bleach and milli‐Q water between each sample. The mock communities were crushed directly in their tube using an eppendorf micropestle. A subsample of the crushed sediment (between 210 and 250 mg of powder) and the crushed mock community sample were processed through the QIAgen DNeasy® PowerSoil® Pro extraction Kit (Germany). To improve tissue lysis, we added 80 μl of Proteinase‐K to 720 μl of CD1 buffer and incubated the sample overnight at 37°C with 400 rpm in a ThermoMixer® (eppendorf AG, Germany). We eluted the DNA in 50 μl of Milli‐Q water (Merck, Kenilworth, USA).

To compare bulk‐DNA from the sediment sample itself and environmental DNA (eDNA) from the ethanol in which the sediment was preserved for 4 years, we subsampled 750 μl of the reserved ethanol for each sample (*n* = 10). The ethanol evaporated in a Concentrator plus (eppendorf AG, Germany) for 2 h and these samples were processed through the QIAgen DNeasy® Blood and Tissue extraction kit (Germany). Again, to improve cell lysis, we added 20 μl of Proteinase‐K to 180 μl of ALT buffer and let the lysis occur overnight at 56°C with 300 rpm in a ThermoMixer®. We eluted the DNA in 50 μl of Milli‐Q water.

### 2.3 Library preparation and sequencing

We amplified the Leray region of the cytochrome c oxidase subunit 1 (COI) with foraminiferal specific primers (Foram_COI_fwd1: 5′‐ GWGGWGTTAATGCTGGTYGAAC ‐3′ and Foram_COI_rev 5′‐ RWRCTTCWGGATGWCTAAGARATC ‐3′) (Macher, Wideman, et al., 2021). The primers were complemented with a Nextera XT tail (Illumina, inc.) in order to label each sample with a unique barcode. Amplifications, library preparation and sequencing of the COI marker were performed according to the protocol from Girard, Langerak, et al. (2022). In short, 2.5 μl of DNA template was mixed to 11.7 μl Milli‐Q water, 2 μl PCR buffer CL 10× (Qiagen), 0.4 μl MgCl2 25 mM, 0.8 μl BSA 10 mg/ml, 0.4 μl dNTP 25 mM, 0.2 μl Taq‐polymerase (Qiagen) 5 U/μl, 1 μl forward primer 10 μM and 1 μl reverse primer 10 μM for a total volume of 20 μl. The PCR1 program was 3 min at 96°C, followed by 40 cycles of 15 s at 96°C, 30 s at 50°C, 40 s at 72°C, followed by 5 min at 72°C. A negative control containing Milli‐Q water instead of DNA template was processed together with the samples for each PCR run to check for potential (cross‐) contamination. We amplified the DNA templates with different dilutions (1×, 10×, 20× and 50×) to optimize the amplification success. The PCR products were cleaned with NucleoMag NGS‐Beads (bead volume at 0.9 times the total volume of the sample, Macherey‐Nagel, Düren, Germany) using the VP 407 AM‐N 96 Pin Magnetic Bead Extractor stamp (V&P Scientific, San Diego, CA, USA). Hereafter, the samples were labeled with the MiSeq Nextera XT DNA library preparation kit (Illumina, San Diego, CA, USA). The PCR2 program was 3 min at 96°C, followed by 8 cycles of 15 s at 96°C, 30 s at 55°C, 40 s at 72°C, followed by 5 min at 72°C. Again, negative controls containing Milli‐Q water instead of DNA template were processed to check for (cross‐) contamination. All blanks were negative. The samples were analyzed with the Agilent 5300 Fragment analyzer with the DNF‐910‐33 dsDNA Reagent Kit (35–1500 bp) protocol (Agilent Technologies, Santa Clara, CA, USA) to confirm successful labeling of the DNA fragments. The samples were pooled together with QIAgility (Qiagen, Hilden, Germany). The final library was cleaned with NucleoMag NGS‐Beads and DNA concentration measured using Tapestation 4150 (Kit HSD 5000, Agilent Technologies, Santa Clara, CA, USA). The sequencing was performed on an Illumina MiSeq V3 PE300 (pair‐end 2× 300 bp) platform at BaseClear B.V. (Leiden, the Netherlands).

### 2.4 Reference database

To improve the already existing COI reference database by Girard, Langerak, et al. (2022), Macher et al. (2022) and Macher, Wideman, et al. (2021), 218 foraminiferal specimens from 21 morphospecies collected at multiple locations were additionally barcoded using single‐cell metabarcoding for this study (see Table S3 for complete reference database used in this study). Before DNA extraction, all specimens were separated and stored in individual eppendorf 1.5‐ml tubes. The specimens were classified to morphospecies level based on the description from Macher, Prazeres, et al. (2021) and Renema (2018), photographed and cleaned in 70% ethanol with a brush and a needle to remove as much non‐foraminiferal material as possible under a stereomicroscope. From DNA extraction, through amplification and sequencing, we followed the protocol from Girard, Langerak, et al. (2022) without modifications. Taxonomic assignments of all species in the reference database (Class, Order and Family levels) follows the latest works from Holzmann et al. (2021), Holzmann and Pawlowski (2017), Macher et al. (2022), Macher, Prazeres, et al. (2021), Pawlowski et al. (2013), Renema (2018), Renema et al. (2001), Siemensma et al. (2017), and the online database WoRMS (https://www.marinespecies.org/). Only the single most abundant amplicon sequence variant (ASV) that was shared by at least two specimens of a species was chosen as reference COI sequence for that species. Four specimens from the genus *Calcarina* and one classified as *Amphistegina lessonii* were assigned their own reference sequence, due to blurry morphological characters, under the condition that it was unique and shared with no other known species used in the database. It is worth noting that five species pairs shared the same ASV (Table S3). The COI reference database now counts 73 sequences, some of which are the same morphospecies from different genetic populations (often from a different locality). Sequences from the COI foraminiferal reference database represent 38 benthic symbiont‐bearing taxa, 38 benthic small heterotrophic taxa and one planktonic taxon.

### 2.5 Data process and quality filtering

Demultiplexed raw reads were merged using the FLASH algorithm (settings minimum overlap = 50, maximum overlap = 300, mismatch ratio = 0.2) (Magoč & Salzberg, 2011) and primers were trimmed with cutadapt (minimum bases that need to match = 10, maximum allowed error rate = 0.2, minimum read length = 10) (Martin, 2011). Sequences with bases below a quality score of 20 were filtered out with Usearch (function ‐fastq_truncqual 20) (Edgar & Flyvbjerg, 2015). To retain only foraminiferal sequences, we filtered out sequences outside the read size range 315–325 bp using PRINSEQ (Schmieder & Edwards, 2011). This filtering step is based on the size range of COI sequence length in our reference database (i.e., 321–323 bp) and therefore limits noise from nonforaminiferal DNA in our dataset. The sequences were then clustered into amplicon sequence variants (ASVs) using UNOISE (settings alpha = 4.0, minimum abundance before clustering = 8) (Edgar, 2016). The output ASV table was further filtered at the cutoff of 0.01%, which removes the ASVs detected with 1 read in a sample counting 10,000 reads, to account for tag switching, sequencing errors or possible (cross‐) contamination. Finally, we assigned taxonomy to the ASVs using blastn against our foraminiferal COI reference database, with the constraints of a query coverage percentage cutoff at 90% and an identity percentage cutoff at 75%. All ASVs not assigned to the Foraminifera against our reference database were blasted against GenBank using the same coverage and identify percentage cutoff.

### 2.6 Data analysis

Molecular taxonomic identity thresholds were defined based on our foraminiferal COI reference database according to the morphological taxonomy as described in section 2.5. These thresholds were defined by comparing the different morphospecies and their COI reference in a pairwise identity percentage matrix featuring all COI 73 sequences from our reference database. Thresholds representing our database best were selected as follows: 75% Phylum, 80% Class, 84% Order, 96% Family, and 99.4% Species (Figure S1). Our reference database did not allow us to confidently set a threshold at the genus level because of the lack of taxa in our reference database within the same genus. These thresholds are subject to change with a growing reference database. We use the thresholds to analyze the mock communities, bulk‐DNA and eDNA samples. Whenever foraminiferal species community compositions were analyzed, only ASVs assigned to >99.4% identity (species level) were considered.

To evaluate the similarity of the replicate at a sampling site and within mock communities as well as the dissimilarity between sampling sites and between mock communities, we calculated the distribution of the mock communities and the samples using nonmetric multidimensional scaling (NMDS). Using distance matrices, we assess whether the distances between groups (here sites and mock communities) were greater than within groups (between technical replicates) with the Analysis of Similarity (ANOSIM; function anosim() from the R package “vegan” (Oksanen et al., 2007)). We used a one‐way Permutational multivariate Analysis of Variance (PermANOVA; function adonis2() from the “vegan” package) to evaluate whether Bray–Curtis distances between groups (here sampling sites, locations, habitat, and sample types) differ. We also performed a multifactorial PermANOVA to consider for interactions between the studied factors, with the sample type and the habitat being fixed factors, and the site a nested factor within locations. Each site has its own habitat (either reef flat or reef slope); therefore, there are no interactions between these two factors. The multifactorial PermANOVA was conducted on the whole dataset and on the dataset restricted to LBF species, because other foraminifera were not considered in the morphological samples. A rarefaction curve analysis was also performed to assess the level of replication required to characterize the diversity at a sampling site (function rarecurve() from the “vegan” package).

Additionally, bulk‐DNA samples from sediments were compared to large benthic symbiont‐bearing foraminifera (LBF) community composition reported from the same sites and same time, as published in Girard, Estradivari, et al. (2022) and Girard, Langerak, et al. (2022). Our samples were collected within 30 m of the samples reported in Girard, Estradivari, et al. (2022) and Girard, Langerak, et al. (2022) at each sampling site. The living LBF community composition was manually assessed: LBF were manually sorted with a stereomicroscope, species were morphologically identified and subsequently counted. The community composition of these samples is later referred to as community compositions morphologically assessed (“morphological samples”). NMDS, ANOSIM, and PermANOVA were also used to assess the similarity between LBF communities from morphological and bulk‐DNA samples. To compare bulk‐DNA and morphological samples adequately, we grouped sequence reference names to match the ones published in Girard, Estradivari, et al. (2022) and Girard, Langerak, et al. (2022) dataset. For example, the DNA reference “*Amphisorus*/*Amphisorus* SpL/*Amphisorus* SpS” and “*Amphisorus* SpS” from the published morphological dataset were renamed to “*Amphisorus* spp.” (see the complete list of combinations Table S4).

## 3 RESULTS

To show the reliability of COI metabarcoding to retrieve foraminiferal community composition, we used six mock communities, bulk‐DNA from 10 sediment samples and eDNA from their ethanol preservative. We amplified all mock communities and samples multiple times (up to seven technical replicates) to check for potential amplification and sequencing biases. The retrieved number of raw reads from the sequencing run summed to 8,969,281 reads. After quality filtering of the raw data, we retained 6,373,423 reads equaling 71.05% of the total raw reads (see Table S5 for details on read number per samples). After applying the cutoff of 0.01% of total reads per sample on the ASVs, 1070 ASVs were retained for further analyses. We compared the ASVs retained against our foraminiferal COI reference database. A total of 1019 ASVs were assigned to Foraminifera (95.2%) (Table 1), nine ASVs were assigned to other phyla (i.e., one to Arthropoda, three to Chordata, two to Discosea, one to Mollusca, one to Platyhelminthes, and one to Tubulinea) (0.8%) and 42 ASVs remained unassigned (4.0%).

**TABLE 1:** Number of foraminiferal amplicon sequence variants (ASVs) unassigned and assigned to different taxonomic levels (Phylum, Class, Order, Family, and Species) and the proportion of total reads it accounts for (%).

	Mock communities		Bulk‐DNA		eDNA	
	ASV	Reads (%)	ASV	Reads (%)	ASV	Reads (%)
Total number of ASVs	155	100	723	100	609	100
Unassigned ASVs	0	0	29	0.2	15	0.4
Foraminifera (>75% ID)	155	100	689	99.7	588	99.5
Class (>80% ID)	155	100	657	99.6	528	98.6
Order (>84% ID)	155	100	622	99.5	474	97.2
Family (>96% ID)	146	97.5	415	91.8	303	79.2
Species (>99.4% ID)	42	79.9	42	59.1	33	34.9
Annotation of the ASVs at the species level	Corresponding to 17 different species from our reference database		Corresponding to 29 different species from our reference database		Corresponding to 24 different species from our reference database	

*Note*: ID, identity percentage.

### 3.1 Metabarcoding workflow success rate

Mock community technical replicates (*n* = 5 per mock community) were nearly identical, both in the retrieved ASVs and their respective number of reads. Around 27% of all ASVs retrieved from the mock communities were classified at the Species level (>99.4% ID) accounting for 79.9% of all reads (Table 1). Technical replicates within each mock community were significantly more similar to each other than between mock communities, (ANOSIM: *p* = .001 and *R* = 1) (Figure 1b).

Bulk‐DNA amplified best with 50× diluted templates, with a total of five to seven technical replicates per bulk‐DNA sample. eDNA samples amplified best with 10× dilution templates; however, one eDNA sample (E‐UPG82‐rf1) did not amplify despite the different dilution trials. We amplified at least three technical replicates for the other nine eDNA samples. Bulk‐DNA and eDNA did not group well together in the NMDS ordination (Figure S2a). Considering all ASVs in the dataset, technical replicates from the bulk‐DNA samples were very similar within the sampling sites and differences between the sites were significant (one‐way PermANOVA [grouping per sampling site]: *R* ^2^ = 0.9868, *F*‐value = 424.03, *p* = .001) (Figure 1c). All 30 most abundant ASVs (accounting for >85% of the total read number per sample) were shared between at least two technical replicates of a bulk‐DNA sample, and 28 of the 30 ASVs were shared between at least three replicates (Table S6). Additionally, the three first replicates covered between 73 and 97% of the diversity (i.e., of the total number of ASVs) of each bulk‐DNA sample (Figure S5).

**FIGURE 2:**
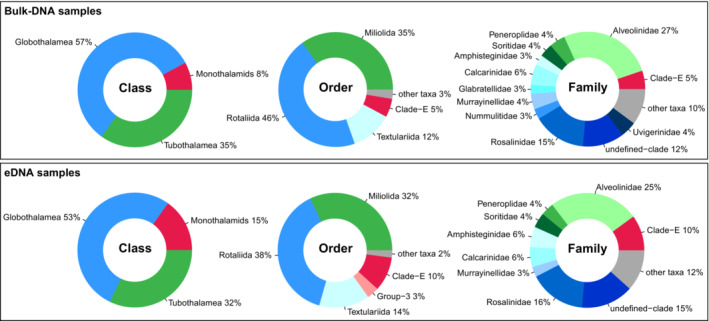
Bulk‐DNA and eDNA foraminiferal community compositions (proportions of the number of ASVs) based on blast hits of our local foraminiferal reference database >75% ID referring to Table 1. “Other taxa” are all ASVs that were in lower abundance than 2% of the total number of foraminiferal ASVs in the community.

Replicates from eDNA samples showed more dissimilarity and spread more extensively in the ordination despite significant differences between the sampling sites (one‐way PermANOVA [grouping per sampling site]: *R* ^2^ = 0.8163, *F*‐value = 19.99, *p* = .001) (Figure S2b). Around 5% of all ASVs were assigned at the species level in both bulk‐DNA and eDNA sample types; however, this portion covered nearly 60% of all reads in the bulk‐DNA and only 35% of the reads in eDNA samples (Table 1). ASVs assigned to monothalamids were two times higher in the eDNA samples thanin the bulk‐DNA samples, but community was dominated by Rotaliida and Miliolida at more than 70% of the ASVs in both bulk‐DNA and eDNA samples (Figure 2). Community composition from bulk‐DNA samples covered 12 Foraminifera families, whereas eDNA samples only nine (Figure 2). Because eDNA samples showed more variability within their technical replicates than bulk‐DNA samples and had significant differences in community composition (including decreased species richness) compared with their bulk‐DNA counterparts (one‐way PermANOVA [grouping per sample type]: *R* ^2^ = 0.0591, *F*‐value = 6.53, *p* = .001) (Figures S2 and S3, Table S7), eDNA samples were not further analyzed in later comparisons.

### 3.2 Species detection from the mock communities

**FIGURE 3:**
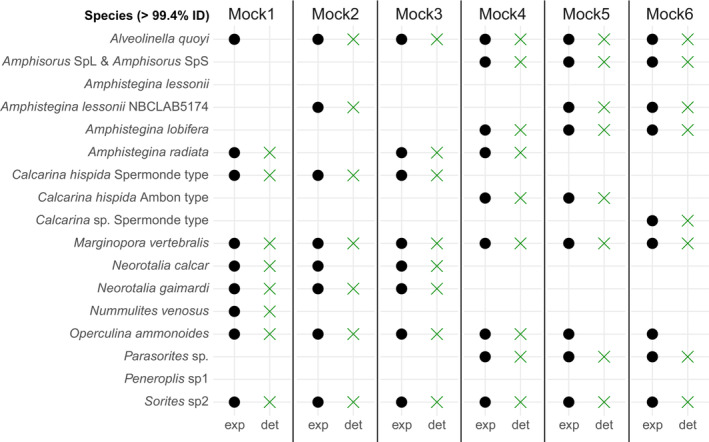
Mock community compositions. Assigned species to ASVs with hits of >99.4% pairwise identity. Expected (“exp”) and detected (“det”) species in each mock community are marked with a back circle and a green cross, respectively. Species are categorized as detected if they were present in more than 50% of the replicates. See Figure S4 for reads detail in each replicates.

Most expected species were detected (present in at least three replicates) in all replicates in all mock communities (Table 2, Figure 3). Mock communities 3 and 4 detected all species in all replicates, whereas the four other mock communities failed to detect one of the species in three or more replicates. *Operculina ammonoides* was the species with the lowest detection rate, missing in two, three and five replicates of the mock communities 2, 6 and 5, respectively (Figure S4, Figure 3), and therefore not detected in mock communities 5 and 6. *Alveolinella quoyi* failed to be detected in mock community 1 and *Neorotalia calcar* from mock community 2. Only expected species were detected in the mock communities, however unexpected ones were also present in very low abundance in one or two replicates (Figure S4, Table 2).

**TABLE 2:** Success rate in mock communities in detecting expected species.

	Mock1	Mock2	Mock3	Mock4	Mock5	Mock6
Number of expected species detected in all replicates	8/9	6/8	8/8	9/9	7/9	8/9
Number of expected species detected in 3 or more replicates (>50%)	8/9	7/8	8/8	9/9	8/9	8/9
Number of replicates detecting all expected species	0/5	1/5	5/5	5/5	0/5	2/5
Number of replicates with erroneous species	1/5	2/5	2/5	1/5	1/5	2/5

### 3.3 Comparison between morphological identification and bulk‐DNA

**FIGURE 4:**
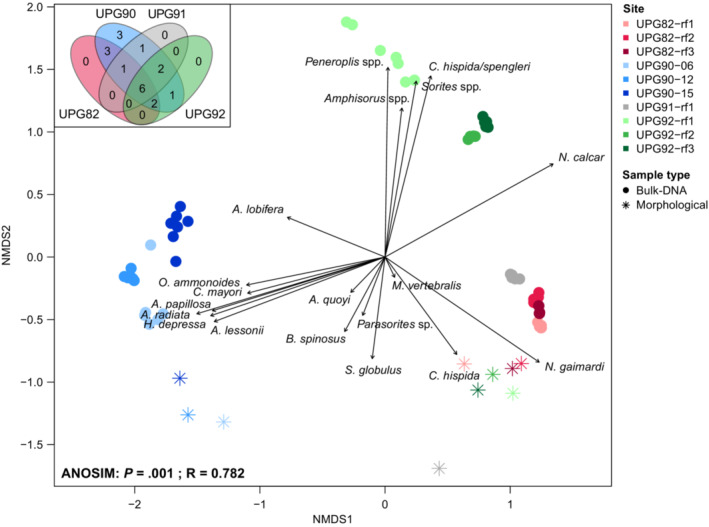
Nonmetric dimensional scaling representation of the LBF community composition of morphological samples and bulk‐DNA from the sediment (including only ASVs assigned at 99.4% ID). A Venn diagram showing the number of shared LBF species between the sampling sites is displayed at the top left corner. Analysis of similarity (ANOSIM, grouping per site) result is displayed at the lower left corner. Morphological samples are marked with an asterix, bulk‐DNA as full circles.

**FIGURE 5:**
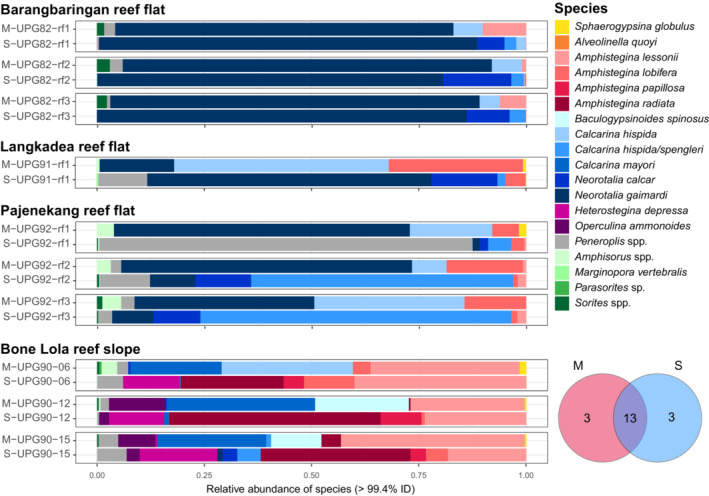
Comparison of large benthic foraminiferal community composition at the species level between the morphological specimen counts (sample numbers with a “M”) and bulk‐DNA from sediments (sample numbers with a “S”) at each sampling sites. A Venn diagram showing the number of species detected in each sample type is displayed in the lower right corner.

To assess success of species detection in our bulk‐DNA from sediments, we compared our results with those of a previous study reporting the large benthic symbiont‐bearing foraminifera (LBF) community composition at the same sampling sites, referred to as “morphological” samples (Girard, Estradivari, et al., 2022). Overall, the morphological samples grouped well with the expected environment, where morphological and bulk‐DNA samples from the same habitat were more similar to each other than those from a different habitat, based on the NMDS ordination (Figure 4). However, within a same habitat, morphological samples were significantly different to the molecular ones, based on the one‐way PermANOVA analysis (Table S7, Figure 4), most likely due to misclassification or overlooking the smaller fraction. Nevertheless, bulk‐DNA and morphological samples were consistently different across habitats (see Table S7). Molecular samples from the reef flat of Pajenekang (UPG92) and Barangbaringan (UPG82) clearly separated in the ordination (one‐way PermANOVA [grouping per island]: *R* ^2^ = 0.7223, *F*‐value = 85.83, *p* = .001), a pattern not so obvious from the morphological samples (one‐way PermANOVA [grouping per island]: *R* ^2^ = 0.6675, *F*‐value = 8.03, *p* = .1) (Figure 4). The multifactorial PermANOVA results show that the factors compared here (i.e., sample type, habitat, sites) are not independent, which is expected due to the experimental design (foraminiferal communities from different sample types originating from the same sampling sites) (see Table S8). However, the groups within these factors (e.g., bulk‐DNA vs Morphology within the sample type) remain significantly different from one another for each multifactorial PermANOVA performed. Similar species richness (S) was detected in bulk‐DNA (*S* = 8) and morphological samples (*S* = 9) (Figure S3). It is worth noting that we have no reference sequences for two species reported by Girard, Estradivari, et al. (2022) and Girard, Langerak, et al. (2022): *Sphaerogypsina globulus* and *Baculogypsinoides spinosus*. Morphological and bulk‐DNA samples shared 13 out of 19 LBF species (Figure 5).

**FIGURE 6:**
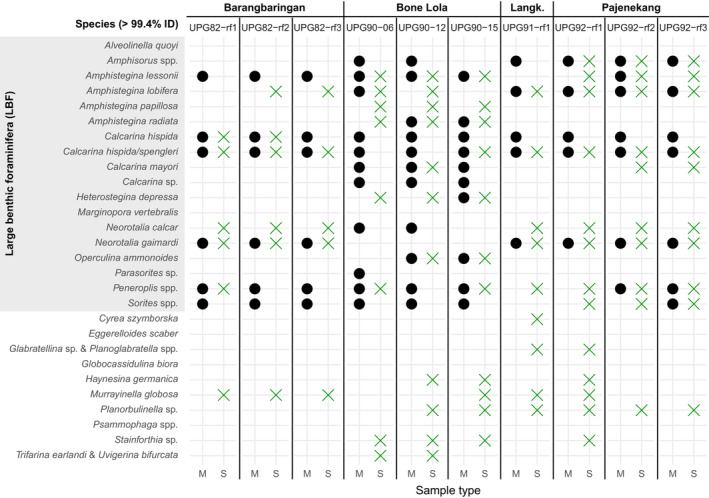
Morphological (M) and bulk‐DNA from sediments (S) detected community compositions. Assigned species to ASVs with hits of >99.4% identity. Expected species (black circles) refer to the ones in morphological samples reported previously in Girard, Estradivari, et al. (2022) and Girard, Langerak, et al. (2022). Detected species in bulk‐DNA samples are marked with a green cross. Species are categorized as detected if they were present in more than 50% of the replicates.

Looking into the LBF community composition in more detail, our results showed that the molecular approach detected a community with sometimes different species composition and sometimes more species (Figures 5 and 6). The diversity of *Amphistegina* spp. reported from the morphological samples was lower on the reef slope of Bone Lola (UPG90) compared with what we found in the bulk‐DNA from the sediments. The same goes for the diversity of Calcarinidae on the reef flat of the three sampling locations, where additional species (e.g., *Neorotalia calcar*) were detected in bulk‐DNA from the sediment samples (Figure 5). Furthermore, the members of the family Soritidae (*Amphisorus* spp., *Parasorites* sp. and *Sorites* spp.), although bigger in size than the other LBF species but less abundant, soritids were not as well reflected in the diversity reported previously. For example, *Sorites* spp. were detected at Pajenekang but not previously reported on the reef flat of this island, and not detected at Barangbaringan and Bone Lola where they were in fact previously reported (Figures 5 and 6).

Besides LBF, a variety of smaller foraminiferal species (>99.4% ID) from the monothalamid and textularid groups were detected in the bulk‐DNA samples (Figure S3, Figure 6). For example, the heterotrophic foraminifera *Murrayinella gibosa* was detected in six bulk‐DNA samples, and *Planorbulinella* sp. in seven bulk‐DNA samples. Others were mainly detected on the reef slope samples, such as *Stainforthia* sp. It was impossible to assign more ASVs to the species level due to the limits of our reference database.

## 4 DISCUSSION

Using mock communities, bulk‐DNA from sediment samples and eDNA from their ethanol preservative, we show the potential of foraminiferal COI metabarcoding to assess reliably foraminiferal community composition. We compared our results with previously reported large benthic foraminiferal (LBF) community composition from the same sites. The COI metabarcoding workflow was successful in retrieving the expected community composition from the bulk‐DNA samples and the mock communities with our self‐built COI reference database. Additionally, we detected a higher number of species in the bulk‐DNA, both in the heterotrophic foraminiferal group and in the LBF group, compared with what Girard, Estradivari, et al. (2022) and Girard, Langerak, et al. (2022) reported. Moreover, bulk‐DNA metabarcoding also enabled the identification of potentially misclassified species difficult to distinguish by nonexperts, for example, *Amphistegina* spp. and *Calcarina* spp.

### 4.1 Bulk‐DNA metabarcoding to improve community composition assessment

Performing metabarcoding on bulk‐DNA from sediment samples showed more reliable results than using eDNA from the ethanol preservative. Likewise, Derycke et al. (2021) concluded that the detection of macrobenthos diversity with bulk‐DNA was significantly higher than with eDNA, explaining that the diversity found in eDNA samples was linked to the morphological traits of the species (Derycke et al., 2021). Another study faced multiple issues while trying to assess the true insect diversity using eDNA from the ethanol preservatives of their samples (Zenker et al., 2020). For bulk‐DNA samples, we suggest to amplify three technical replicates to characterize adequately the diversity and increase resolution at a given sampling site (van der Loos & Nijland, 2020 and references herein).

Bulk‐DNA metabarcoding has the advantage of detecting not only the target species group (e.g., LBF), but also the small, heterotrophic foraminifera fraction living in the sediment. This was also reported by Frontalini et al. (2020) using a ribosomal marker. Indeed, morphologically, it is more feasible to sort and assess the community composition of hard‐shelled foraminifera, while generally smaller, soft‐shelled, and naked foraminifera are often overlooked. Bulk‐DNA metabarcoding has the power to highlight their presence to a certain taxonomic level, despite remaining gaps in the reference databases. At this stage, bulk‐DNA metabarcoding is a promising tool to assess presence–absence of species, as shown in this study. Quantitative information such as the number of gene copies could offer a more in‐depth understanding of how molecular datasets translate to ecological patterns and community dynamics. However, our results show little congruence between the number of counted specimens and number of COI gene copies per species. Ribosomal gene copies have also been found to differ drastically between species, with no correlation to specimen size (Milivojević et al., 2021; Weber & Pawlowski, 2013). Future research should assess the suitability of the COI marker region for abundance‐based analyses.

### 4.2 Limitation of the actual COI foraminiferal reference database

Compared with the 18 S rRNA foraminiferal reference database that has about 1100 nonredundant species (Guillou et al., 2013), our COI reference database is limited to 77 species because the gene was only recently sequenced for the first time (Macher, Wideman, et al., 2021). Despite that most of our sequences (95%) could be assigned to the phylum Foraminifera, merely 5% of all sequences in our dataset were assigned to the species level. We expect the detectable foraminiferal species diversity of our DNA samples to increase with a growing number of references. The COI region we used as a marker is conserved and therefore the percentage identity at the species level is very high (99.4%). Some species, however, cannot be differentiated based on this ca. 320 bp. fragment. These species couples are, for example, *Calcarina hispida* from Ambon (Indonesia) and *C. spengleri*, *Neoratalia gaimardi* and *Baculogypsina sphaerulata*, additionally to *Amphisorus* SpL and SpS, which have clear morphological differences, but are genetically hard to distinguish also with other molecular markers (Macher, Prazeres, et al., 2021). A longer COI fragment that would cover an additional, more variable region of the mitochondrial genome could possibly solve the classification problem. Nonetheless, the COI marker works phylum wide and is very specific to Foraminifera, avoiding co‐amplification of the foraminiferal microbiome and other non‐target organisms present in the sediment or water. Therefore, the COI marker tested in this study is promising for large scale foraminiferal community metabarcoding.

### 4.3 Considerations and critical steps for bulk‐DNA metabarcoding

Detection of certain species may fail especially if big and sturdy, or very small, foraminiferal shells are not crushed and homogenized properly. This step is therefore critical and should be carefully performed before extraction of bulk‐DNA from the sediment samples. Number of replicates and sequencing depth is also an important factor to consider when processing a high number of sediment samples (van der Loos & Nijland, 2020 and references herein). Technical (PCR) replicates used in our study showed that no significant bias was introduced by amplification and sequencing. However, samples expected to have species in low abundances and very small specimens would benefit from at least three replicates in order to be detected. Additionally, in our study, we detected small taxa with a number of reads as low as three. Therefore, more than 135 bulk‐DNA samples (including replicates) on an Illumina MiSeq 300PE run might be too many to retrieve the full diversity of the community. Other sequencing devices, such as Illumina NovaSeq, would be more appropriate for large numbers of samples and robust diversity in order to be cost‐efficient (Singer et al., 2019).

At this stage, our study confirmed that the bulk‐DNA metabarcoding workflow using the foraminiferal COI marker adequately retrieved the diversity and community composition of both the mock communities and bulk‐DNA samples, especially regarding presence–absence results. With its decreased variability, the COI marker renders bulk‐DNA metabarcoding a powerful tool to assess more efficiently and reliably foraminiferal community composition, under the condition that the reference database is adequate to the target taxa. Bulk‐DNA metabarcoding would be a great addition to, for example, reef monitoring programs.

## AUTHOR CONTRIBUTIONS

**Elsa B. Girard:** Conceptualization (equal); formal analysis (lead); methodology (lead); visualization (lead); writing – original draft (lead); writing – review and editing (lead). **Jan‐Niklas Macher:** Conceptualization (supporting); formal analysis (supporting); methodology (supporting); supervision (supporting); validation (equal); writing – original draft (supporting); writing – review and editing (supporting). **Jamaluddin Jompa:** Resources (equal). **Willem Renema:** Conceptualization (equal); formal analysis (supporting); methodology (supporting); resources (equal); supervision (lead); validation (equal); visualization (supporting); writing – original draft (supporting); writing – review and editing (supporting).

## CONFLICT OF INTEREST

The authors report no conflicts of interest. The authors alone are responsible for the content and writing of the paper.

## Supporting information


Tables S1–S8

Figures S1–S5

## Data Availability

Genetic data: The new sequences added to our COI reference database are available on GenBank (https://www.ncbi.nlm.nih.gov/genbank/, accession numbers: ON352777‐ON352803). Raw data and metadata from the mock communities, bulk‐DNA and eDNA samples were uploaded to Sequence Read Archive ‐ SRA (https://www.ncbi.nlm.nih.gov/, BioProject PRJNA827111). Analysis data: All R codes and datasets used in this study were deposited on GitHub platform (https://github.com/EBGirard/COImetabarcoding). Benefits generated: Benefits from this research accrue from the sharing of our data and results on public databases as described above.
