# Supplementary material for: COI metabarcoding of large benthic Foraminifera: Method validation for application in ecological studies
Source: Ecol Evol. 2022 Nov 22;12(11):e9549. doi: 10.1002/ece3.9549 (PMC9682090; doi:10.1002/ece3.9549)
Supplement: Supplementary file 2 — Figures S1–S5 [file ECE3-12-e9549-s002.docx]

**Supplemental Information for:**

**COI metabarcoding of large benthic Foraminifera: method validation**

**for application in ecological studies**

Elsa B. Girard^a,b^, Jan-Niklas Macher^a^, Jamaluddin Jompa^c^, Willem Renema^a,b^

a Naturalis Biodiversity Center, Darwinweg 2, 2333 CR Leiden, the Netherlands

b IBED, University of Amsterdam, Sciencepark 904, 1098 XH Amsterdam, the Netherlands

c Marine Science Department, Faculty of Marine Science and Fisheries, Hasanuddin University, Jl.

Perintis Kemerdekaan Km. 10 Tamalenrea, Makassar 90245, Indonesia

**Table of Contents:**

| **Supplementary Figure S1** | Page 2 |
| --- | --- |
| **Supplementary Figure S2** | Page 3 |
| **Supplementary Figure S3** | Page 4 |
| **Supplementary Figure S4** | Page 5 |
| **Supplementary Figure S5** | Page 6 |
| **Captions supplementary tables** | Page 7 |

1. **Supplementary figures**


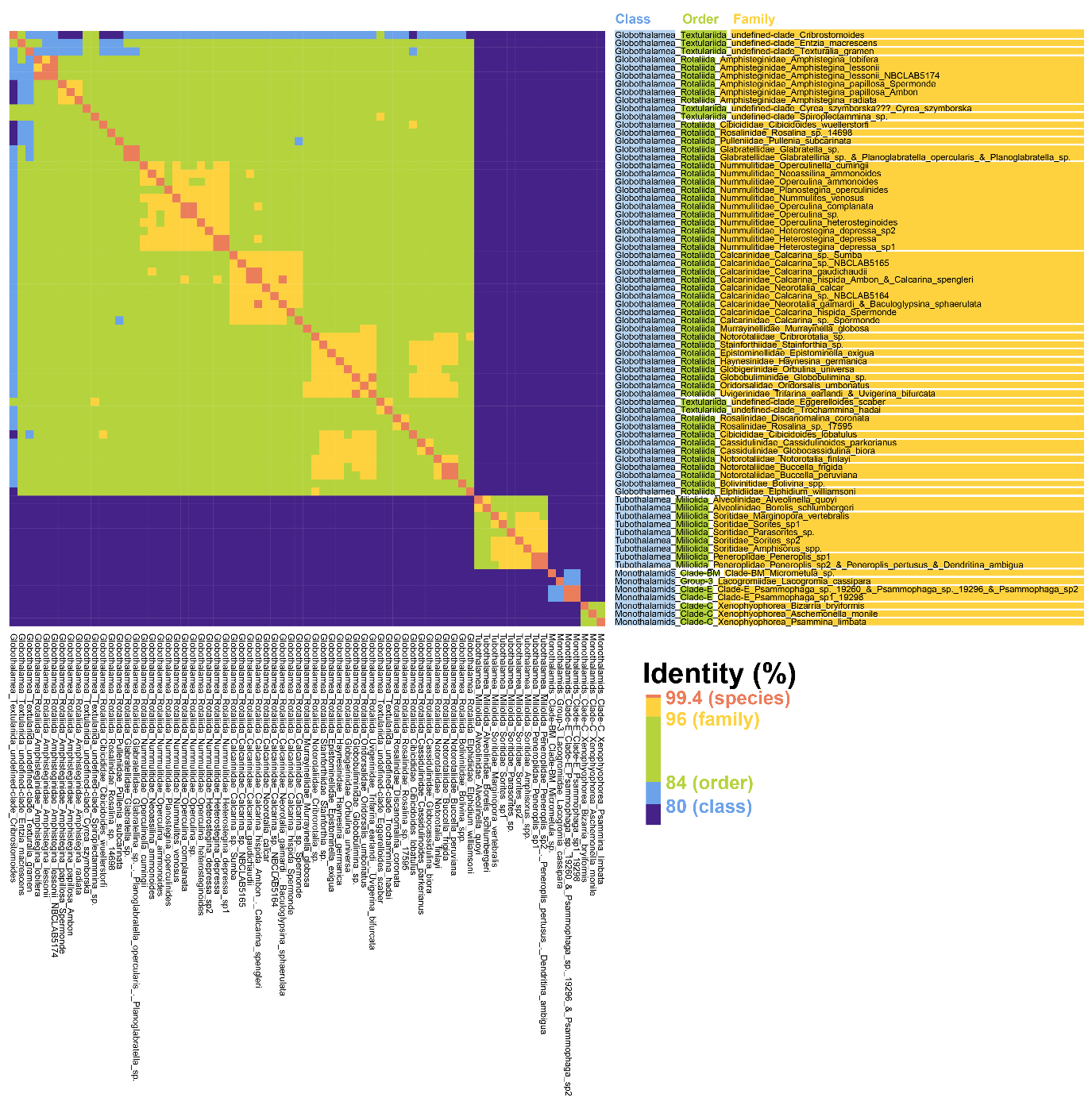


*Supplementary Figure S1. A) Identity percentage threshold used to fit our COI foraminiferal reference database to the following: 75% Phylum, 80% Class, 84% Order, 96% Family and 99.4% Species.*

*
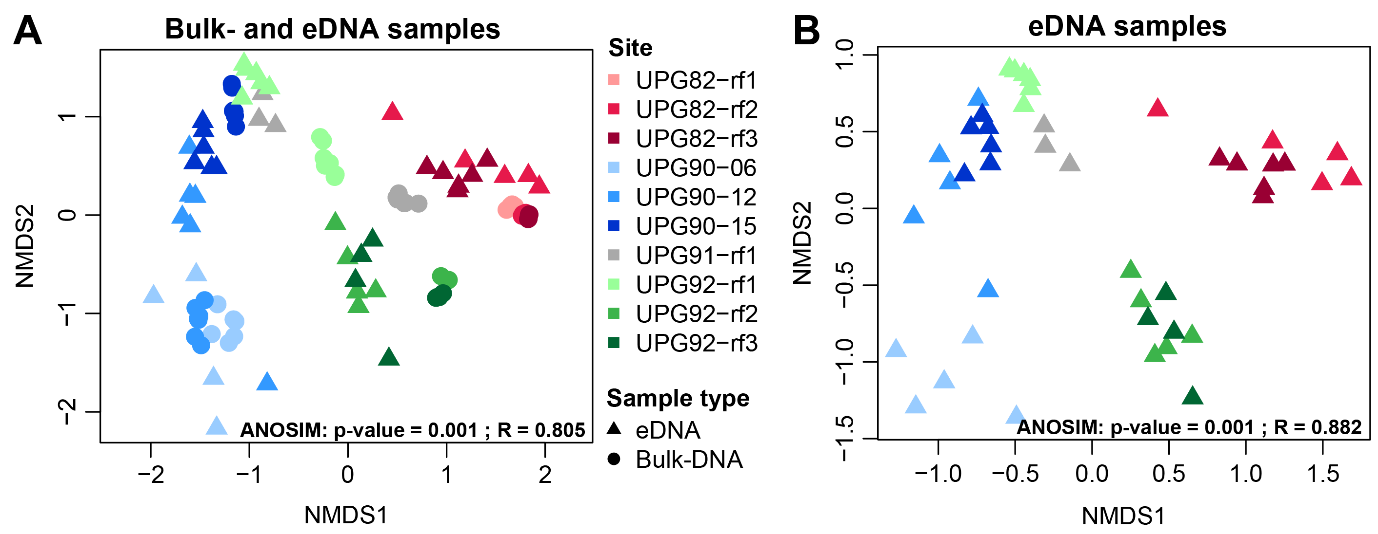
*

*Supplementary Figure S2. Non-metric dimensional scaling representation of the bulk-DNA and eDNA samples (A) and eDNA samples from the ethanol preservative alone (B), including all their replicates and all ASVs. Analysis of similarity (ANOSIM) results are indicated at the lower right corner of each graph (grouping per site). Sampling sites are marked by different colours.*


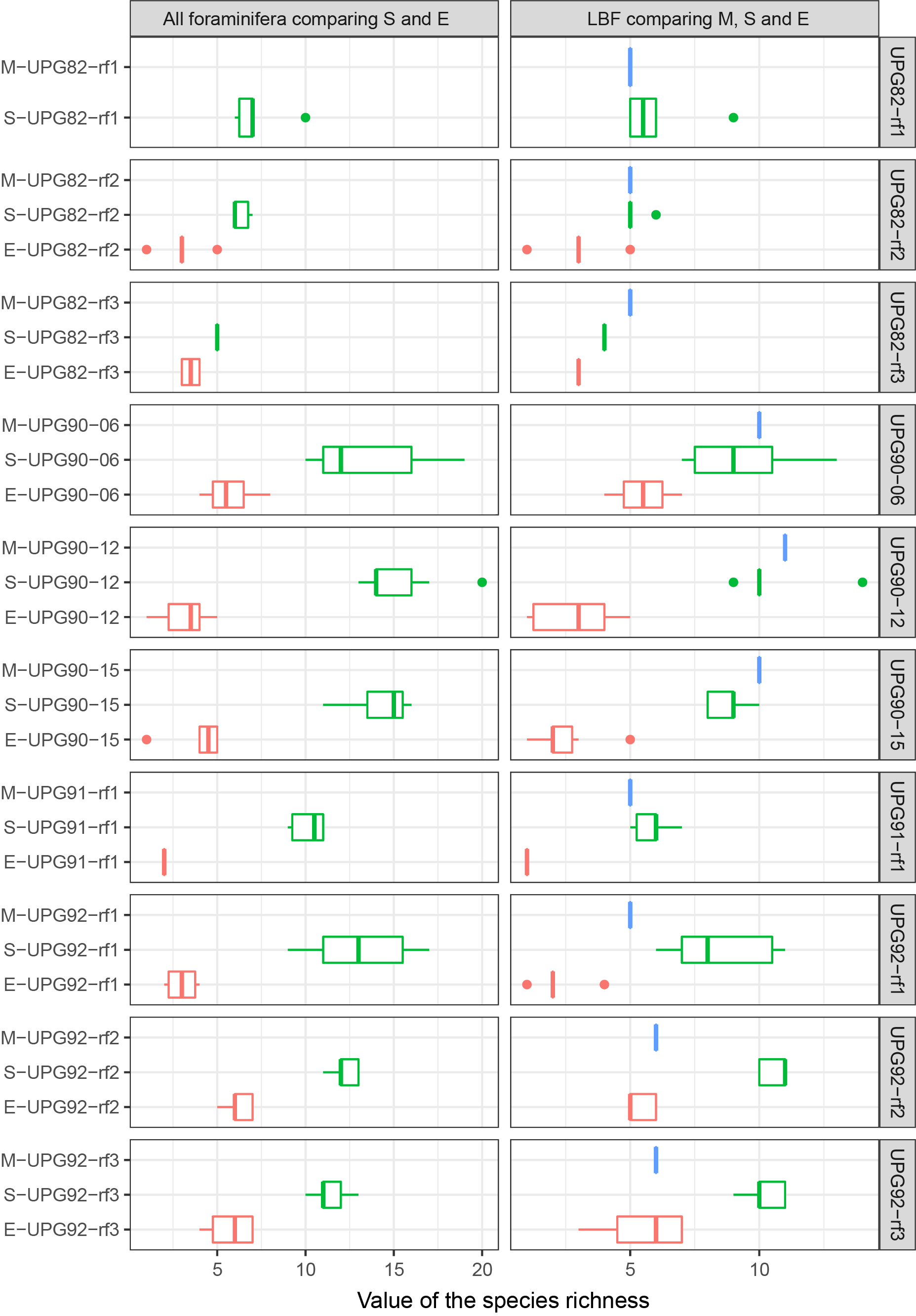


*Supplementary Figure S3. Species richness assess for each sample type at each sampling location. On the left panel, the complete foraminiferal assemblage composition from bulk-DNA (S) and eDNA (E) samples are compared. On the right panel, only the LBF assemblage composition from morphological (M), bulk-DNA and eDNA samples is compared. Morphological samples in blue, bulk-DNA samples in green and eDNA samples in red.*

*­*


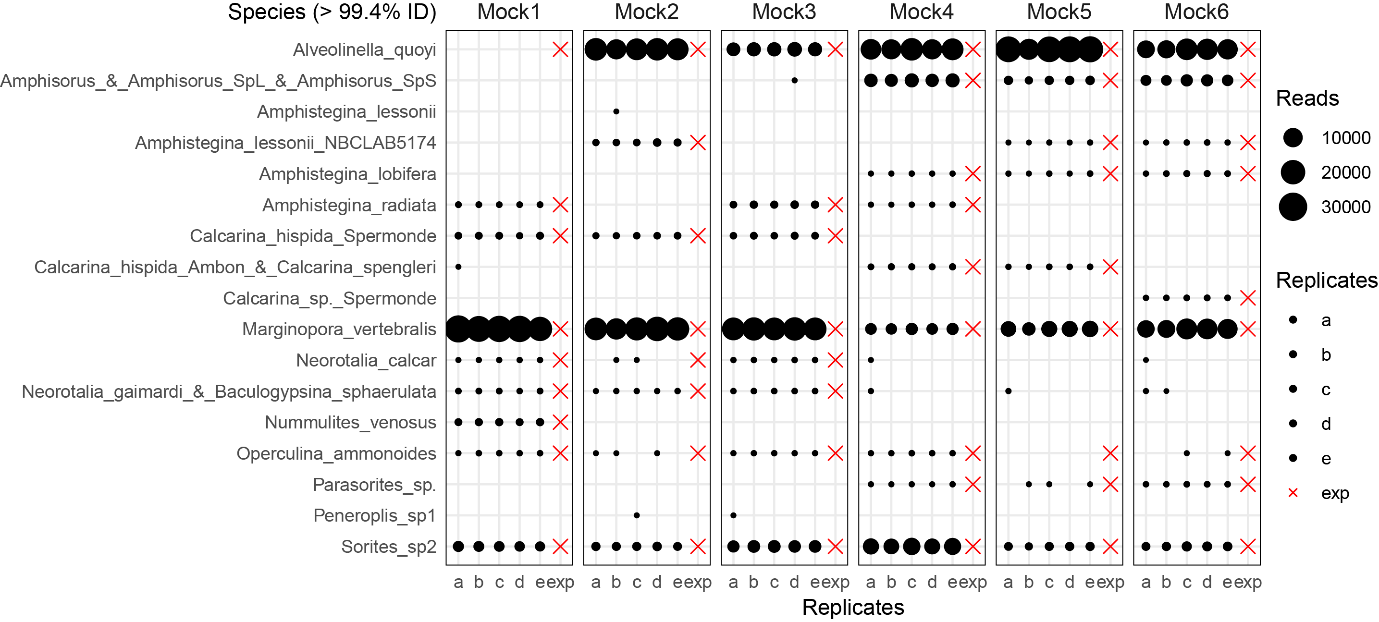


*Supplementary Figure S4. Number of reads per species presented for each replicate of each mock community. Black circles are scaled to the number of reads. Absence of a circle means that no ASV matching the species at > 99.4% ID matched our database and the species is therefore considered absent from the sample. The red “X” represent the expected species present in each mock community, respectively.*


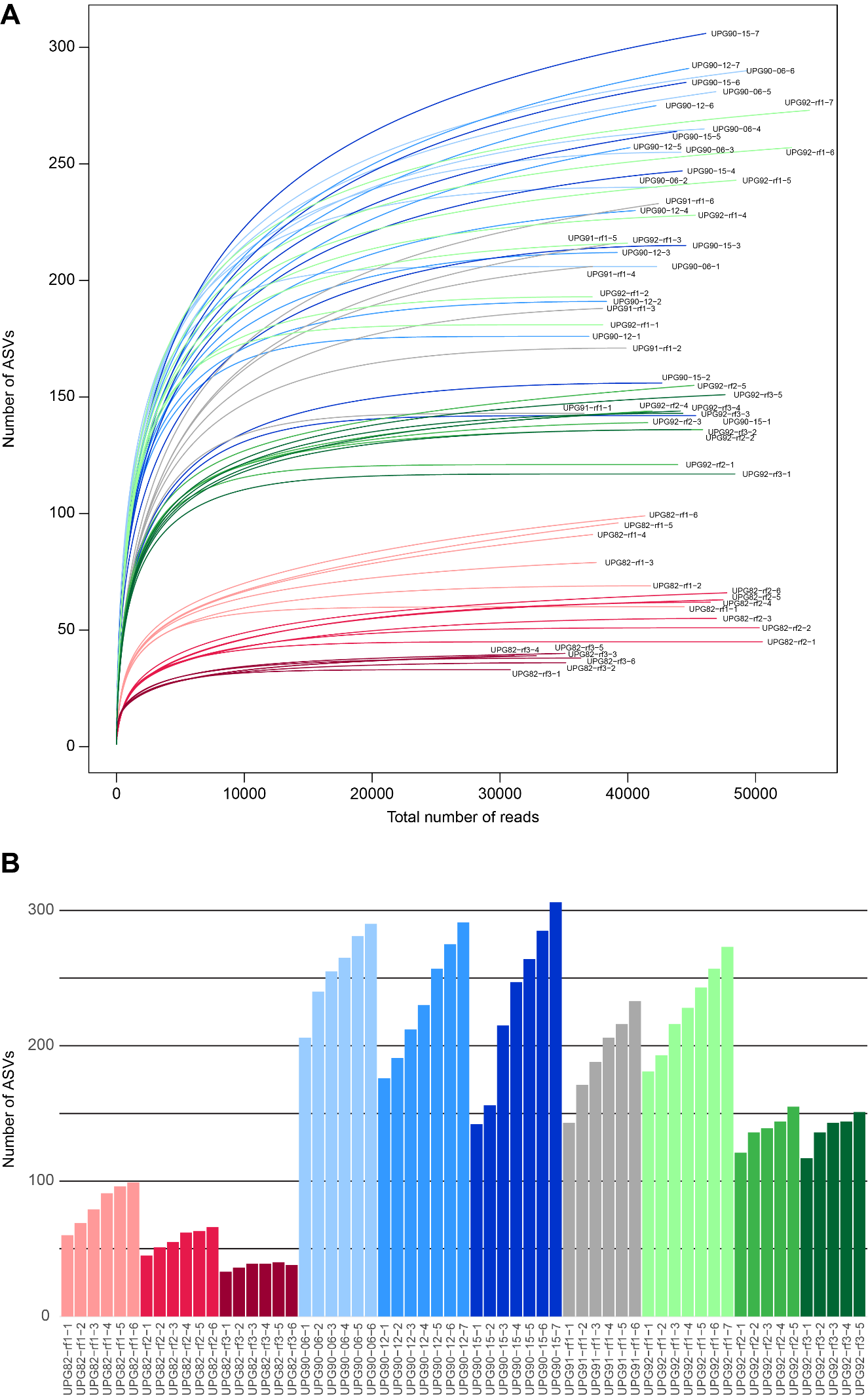


*Supplementary Figure S5. A) Rarefaction curve analysis and B) bar chart showing the number of ASVs cumulated in 1, 2, 3, 4, 5 (and 6, 7) technical replicates for each bulk-DNA samples. More than 70% of the total number of ASVs was retrieved with three replicates.*

1. **Supplementary tables**

*Supplementary Table S1. Metadata on sediment samples.*

*Supplementary Table S2. Mock community composition.*

*Supplementary Table S3. Foraminiferal reference database of Mitochondrial COI used for this study. References added to the database in this study are stated in the column “Reference”. Sequences associated to more than one morphospecies are clearly indicated by having multiple species name in the column “Morphospecies”. References coming from a single specimen are marked with an additional number to the species name (e.g.,* Calcarina *sp. NBCLAB5164 or* Bizarria bryiformis *18256).*

*Supplementary Table S4. List of LBF species used to compare the sediment and EtOH samples with the traditional ones reported in Girard et al. (2022).*

*Supplementary Table S5. Detailed read numbers for all samples at three different step on the filtering process: 1) raw reads from the sequencer, 2) number of reads after filtering but before cutoff and 3) number of reads after cutoff.*

*Supplementary Table S6. Assessment of the number of share ASVs among bulk-DNA technical replicates at different thresholds: selected 10, 20, 30, 40 and 50 most abundant ASVs per bulk-DNA sample.*

*Supplementary Table S7. One-way PermANOVA analysis results with different grouping designs.*

*Supplementary Table S8. Multi-factorial PermANOVA analysis results to assess interactions between factors, including full PermANOVA table for the three dimensional analyses.*
